# Development and Long-Term Follow-Up of an Experimental Model of Myocardial Infarction in Rabbits

**DOI:** 10.3390/ani10091576

**Published:** 2020-09-04

**Authors:** Patricia Genovés, Óscar J. Arias-Mutis, Germán Parra, Luis Such-Miquel, Manuel Zarzoso, Irene Del Canto, Carlos Soler, Ana Díaz, Eva Blanch, Antonio Alberola, Luis Such, Francisco J. Chorro

**Affiliations:** 1 INCLIVA, Institute of Health Research, 46010 Valencia, Spain; pagenomar@gmail.com (P.G.); oscarariasphd@gmail.com (Ó.J.A.-M.); German.Parra@uv.es (G.P.); idelcan@alumni.uv.es (I.D.C.); 2 Department of Physiology, Universitat de València, 46010 Valencia, Spain; Carlos.Soler@uv.es (C.S.); antonio.alberola@uv.es (A.A.); Luis.Such@uv.es (L.S.); 3 Department of Physiotherapy, Universitat de València, 46010 Valencia, Spain; Luis.Such-Miquel@uv.es (L.S.-M.); manuel.zarzoso@uv.es (M.Z.); 4 CIBERCV, Carlos III Health Institute, 28029 Madrid, Spain; 5 Electronic Engineering Department, Universitat Politècnica de València, 46022 Valencia, Spain; 6 UCIM, Universitat de València, 46010 Valencia, Spain; Ana.Diaz@uv.es (A.D.); eva.blanch@uv.es (E.B.); 7 Cardiology Department, Hospital Clinico Universitario, Universitat de València, Avda. Blasco Ibañez 17, 46010 Valencia, Spain

**Keywords:** myocardial infarction, ischemia/reperfusion injury, ventricular remodeling, ventricular arrhythmias, cardiac mapping, experimental surgery

## Abstract

**Simple Summary:**

Ischemic heart disease is one of the leading causes of death. A series of processes occur during acute myocardial infarction that contribute to the development of ventricular dysfunction, with subsequent heart failure and ventricular arrhythmias, which account for most episodes of sudden cardiac death in these patients. These complications are associated with the adverse cardiac remodeling that occurs during the healing process following an acute episode. The remodeling causes the appearance of a substrate that can trigger life-threatening arrhythmias, such as tachycardia and/or ventricular fibrillation. The development of experimental models for analyzing the basic mechanisms involved in the pathophysiology of myocardial infarction enables the study of different therapeutic approaches aimed at improving the patient´s prognosis. The present study describes the methodology and the results obtained in a 5-week chronic infarction (one hour followed by reperfusion) in a rabbit model. The viability of the model, the care provided, the characteristics and extent of the lesions, the inducibility of arrhythmias, and the reproducibility of the methods and results have been analyzed.

**Abstract:**

A chronic model of acute myocardial infarction was developed to study the mechanisms involved in adverse postinfarction ventricular remodeling. In an acute myocardial infarction (AMI), the left circumflex coronary artery of New Zealand White rabbits (n = 9) was occluded by ligature for 1 h, followed by reperfusion. A specific care protocol was applied before, during, and after the intervention, and the results were compared with those of a sham operated group (n = 7). After 5 weeks, programmed stimulation and high-resolution mapping were performed on isolated and perfused hearts using the Langendorff technique. The infarct size determined by 2,3,5-triphenyltetrazolium chloride inside of the area at risk (thioflavin-S) was then determined. The area at risk was similar in both groups (54.33% (experimental infarct group) vs. 58.59% (sham group), ns). The infarct size was 73.16% as a percentage of the risk area. The experimental infarct group had a higher inducibility of ventricular arrhythmias (100% vs. 43% in the sham group, *p* = 0.009). A reproducible chronic experimental model of myocardial infarction is presented in which the extent and characteristics of the lesions enable the study of the vulnerability to develop ventricular arrhythmias because of the remodeling process that occurs during cardiac tissue repair.

## 1. Introduction

Cardiovascular disease, especially ischemic heart disease, is the leading cause of death nowadays [1,2,3,4,5], and global medium-term projections still consider ischemic heart disease as the most frequent [6]. Basic research and its transfer to the clinic are necessary to deepen the knowledge on the causes and mechanisms involved, as well as in the development and improvement of preventive, diagnostic, and therapeutic procedures to detect the disease in the preclinical stages, prevent its manifestations, and/or reduce the adverse effects [7,8,9,10,11,12,13,14].

The use of animal models to study ischemic heart disease and, more specifically, the phenomena that characterize the processes of myocardial ischemia and reperfusion has provided a wide range of information. This information covers the molecular mechanisms and signaling pathways involved in the development of myocardial damage to the local and general regulatory mechanisms that can be modified to protect and limit the adverse consequences of the disease, including remodeling, ventricular dysfunction, cardiac arrhythmias, and sudden death [7,8,10,15,16,17,18,19,20,21,22,23,24]. Sudden cardiac death is a major public health problem. It is the leading cause of death in the United States and exceeds the mortality caused by all types of cancer [25]. Therefore, it is of interest to investigate the mechanisms implicated in its occurrence, which, in most cases, are related to the induction of malignant ventricular arrhythmias, such as ventricular tachycardia (VT) or fibrillation (VF). With respect to the analysis of the arrhythmogenic mechanisms occurring in ischemic heart disease, experimental studies can closely mimic the main characteristics of the human pathology [8]. Experimental models have also provided valid information on the meaning, usefulness, and applications of various diagnostic and therapeutic techniques, including those based on cardiac imaging, recording of electrophysiological activity, and coronary intervention [13,15,26], which are all techniques that can improve patient care when used in clinical practice.

In general terms, the models allow to selectively modify the different variables involved in myocardial damage due to ischemia/reperfusion, as well as enable a rigorous comparison with control cases. Other advantages are the adaptivity of the experimental design to the objectives of the study and the use of direct and precise techniques to obtain the desired information. Nevertheless, there are also limitations derived from interspecies differences and the necessary extrapolation of the obtained results [7,10,11]. The reproducibility of the methodology and experimental conditions, as well as of the results, is another aspect that needs to be considered [10,11,27].

The present study describes an experimental model of chronic (five weeks) myocardial infarction (1 h followed by reperfusion) in rabbits and the electrophysiological behavior of the chronic infarcted heart regarding its vulnerability to develop tachycardia and/or ventricular fibrillation by programmed stimulation. This study aimed to analyze the viability of the model, the care provided to the animals, the characteristics and extent of the lesions, the inducibility of arrhythmias. Moreover, this experimental model can be used to analyze the basic mechanisms involved in the pathophysiology of myocardial infarction, including the ventricular and electrophysiological remodeling that occurs during cardiac tissue repair after the acute episode.

## 2. Materials and Methods

### 2.1. Animals

The animal care and the experimental protocols used in this study complied with the European Union directive 2010/63 on the protection of animals used for scientific purposes and were approved by the Institutional Animal Care and Use Committee of the University of Valencia (2015/VSC/PEA/00233 type 2). Twenty-one 15-week old male New Zealand White rabbits with a mean weight ranging from 3 to 3.5 kg were used. The animals were individually housed in specific cages for rabbits, in compliance with Spanish RD 53/2013 regulations, with environmental and food enrichment and under controlled conditions of temperature (room temperature of 19 ± 2 °C), relative humidity (55 ± 10%), and artificial 12-h light-dark cycles. The animals were fed ad libitum with standard feed and water. The experimental procedure was started after 15 days of acclimatization.

### 2.2. Experimental Design

**Figure 1:**
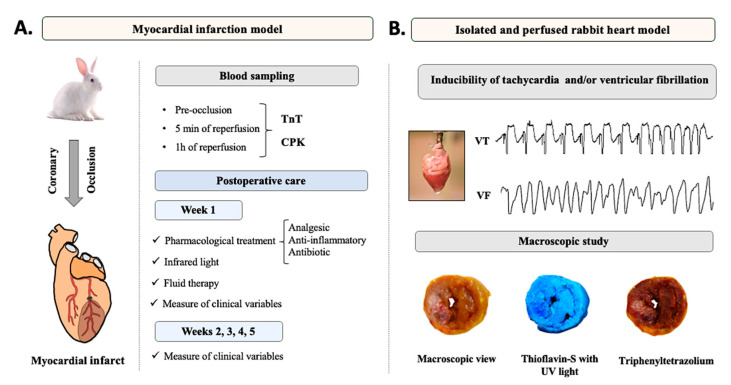
Schematic representation of the experimental design of the present study. (**A**): The chronic experimental procedure of acute myocardial infarction together with the biochemical markers analyzed (troponin T (TnT) and total creatine phosphokinase (CPK)) and a summary of the postoperative care provided during the 5 weeks of the experimental protocol. (**B**): Procedure with isolated and perfused hearts. An electrocardiographic record of induction of a self-limiting ventricular tachycardia (VT) and a ventricular fibrillation (VF) is shown. An example of the stains used in the macroscopic study to determine the infarct size (triphenyltetrazolium) and the area at risk (thioflavin-S) is shown.

The animals were allocated to the sham group (n = 9) and the acute myocardial infarction (AMI) group (n = 13). The same experimental protocol was performed in both groups with the only difference being the myocardial infarction induction in the AMI group. After performing the designed preoperative phase, the animals were subjected to a median sternotomy to induce AMI after locating the left circumflex coronary artery by occluding it for 1 h. Thereafter, the artery was reperfused by removing the occlusion and the chest was closed using standard suture procedures. Blood samples were intraoperatively collected before and after the coronary occlusion. The animals were housed for 5 weeks, and a postoperative care protocol specifically designed for this experimental model was applied during that period. In the sixth week, the animals were euthanized via anesthetic overdose, the heart was removed, and an electrophysiological study was performed in isolated hearts using a Langendorff perfusion system. Thereafter, the hearts were perfused with thioflavin-S and stained with triphenyltetrazolium chloride for macroscopic analysis of the infarct size in relation to the area at risk of the occluded coronary artery and the left ventricle. Figure 1 shows a summary of the experimental design of the present work.

### 2.3. Presurgical Phase

The animals were subcutaneously medicated with analgesics and antibiotics at the following doses: buprenorphine (0.03 mg/kg/6 h), meloxicam (0.2 mg/kg/24 h), and enrofloxacin (10 mg/kg/24 h). In addition, a premedication was intramuscularly administered with an anesthetic and analgesic (ketamine, 35 mg/kg) and a sedative, muscle relaxant, and analgesic (xylazine, 5 mg/kg). A peripheral intravenous line was set up in the marginal vein of each ear. One line was used for the administration of liquids and medications, and the other was used for blood collection.

### 2.4. Anesthesia, Endotracheal Intubation, Measurement of Clinical Parameters, and Surgical Field Preparation

The anesthetic induction was intravenously performed with a bolus of propofol (10 mg/kg) and subsequently maintained with inhalation of isoflurane at 1.5–2% minimum alveolar concentration. With the animal already sedated, the chest was shaved with an electric razor. Thereafter, the animal was endotracheally intubated with a 2-mm tube without a cuff. The animal was placed in a sternal recumbent position with the neck hyperextended at a right angle to the rest of the body so that the mouth, larynx, and trachea were aligned. Once intubated, the animal was connected to a volumetric mechanical ventilator for assisted respiratory function. Once the animal was prepared, it was placed in a supine position on the operating table with the upper and lower limbs extended and fixed to the table. During the surgical procedure, noninvasive multiparametric monitoring was performed, allowing real-time visualization of three-lead electrocardiogram signals, heart rate, noninvasive lower-limb blood pressure, pulse oximetry on the tongue, capnography (respiratory rate and carbon dioxide), and body temperature. During the surgery, the body temperature (39 ± 0.5 °C) of the animals was maintained using thermal blanket as a heating unit. Once positioned, the rabbit was covered with a surgical cloth, exposing only the surgical area, which was disinfected with 10% povidone-iodine.

### 2.5. Surgical Procedure

With the rabbit in a supine position, the median sternotomy was started. A longitudinal central thoracic incision was performed from the manubrium to the xiphoid cartilage. With an electro-scalpel and electrocautery, the incision was deepened until the anterior surface of the sternum was reached. A blunt dissection was performed between the caudal edge of the costal cartilage of rib VIII and the inferior xiphoid process, and a substernal tunnel was created by digital dissection. Subsequently, the sternotomy was performed with Mayo scissors through the substernal tunnel. To expose the heart, an autostatic separator was placed and a pericardiotomy was performed, taking special care not to damage the pleurae. To visualize the left circumflex coronary artery, a surgical stitch was placed on the apex without knots with a 6-0 suture, which allowed to move the heart. After turning the heart counterclockwise and locating the artery, transfixion of the myocardium around the coronary was performed with a 2-0 suture in the first third of the length of the artery, for its subsequent occlusion for 1 h. Coronary occlusion was performed by placing a 5-mm braided silk cord on the artery followed by a ligature over it. This coronary occlusion was successfully performed, as an ST-segment elevation was observed in situ on the vital sign monitor, which correlated with a discoloration of the occluded area. After the occlusion time, the artery was reperfused by removing the ligature. Thereafter, the heart was introduced into the pericardial cradle and the sternotomy was closed using sutures. A thoracic drain was placed by making an incision in the skin in the fourth intercostal space and passing the tube through the subcutaneous tissue into the cavity. Once the tube was in the correct place, it was fixed to the skin using a tobacco-pouch 3-0 resorbable monofilament suture and attached to an underwater seal chamber. The edges of the sternum were then reduced and fixed using a figure of eight suture between the ribs on one side and using a 3-0 absorbable monofilament suture on the other side. The muscle layer was closed by simple discontinuous stitching using a 3-0 resorbable monofilament suture. Finally, the subcutaneous layer was closed with a continuous 3-0 resorbable monofilament suture and, subsequently, the cutaneous layer was closed with an intradermal suture of the same material. Staples were placed to strengthen the skin suture. The wound was then covered with a self-adhesive dressing. After insufflating the lung, the drain was removed with a rapid movement, and the suture was immediately closed in a tobacco pouch shape to hermetically seal the drain duct.

### 2.6. Blood Sampling

To confirm the presence of infarction, the following biochemical markers were analyzed in serum samples: troponin T (TnT) and total creatine phosphokinase (CPK). Three blood samples were collected from the marginal ear vein in both groups. In the AMI group, the first sample was collected after performing coronary artery transfixion and before performing coronary occlusion. Following the reperfusion of the artery, the second sample was collected after 5 min and the third sample was collected after 1 h. In the sham group, the samples were collected at the same time points as in the AMI group. The samples were stored in a tube with separating gel and kept refrigerated (between 4 and 8 °C) until centrifugation. The samples were centrifuged at 4 °C for 15 min at 1500× *g*. The supernatant (blood serum) was collected and stored at −80 °C until use.

### 2.7. Recovery Protocol

The anesthesia was reversed by interrupting the administration of isoflurane. The proper awakening of the animal and the recovery of reflexes for the removal of the mechanical ventilator were checked. Thereafter, oxygen support was maintained through a face mask. The animals were extubated only when the effects of the pharmacological agents were reversed and correct ventilatory mechanics were observed, that is, the animal had spontaneous respiration with a respiratory rate of at least 30 rpm and maintained a O_2_ saturation >95%. During this phase, body temperature was maintained using infrared light (275 W). Subsequently, the animals were individually housed in their respective cages.

### 2.8. Postoperative Care

#### 2.8.1. Week 1

Body temperature was maintained using infrared light (275 W) for 96 h during housing. After the surgery, an analgesic and antibiotic regimen was subcutaneously administered at the following doses: buprenorphine (0.03 mg/kg/6 h/3days), meloxicam (0.2 mg/kg/24 h/7 days), and enrofloxacin (10 mg/kg/24 h/7 days). With respect to the analgesic treatment on the fourth day, a controlled- and continuous-release osmotic pump was subcutaneously implanted and set to a rate of 10 µL/h of buprenorphine (0.03 mg/kg). This pump was removed at the end of the postoperative analgesic treatment. To compensate for the inappetence in all of the recently operated animals, fluid therapy was administered for 3 days through a peripheral intravenous line in the marginal ear vein with a 5% glucose solution at a rate of 42 mL/h for 2 h/day. A physical examination was performed daily to observe the general body condition, attitude, posture, and level of anxiety. In addition, pulmonary and cardiac auscultation was performed. The status of the surgical wound was examined, and the corresponding cures were performed. The digestive functionality was controlled by observing the daily amount of feces, also daily intake was controlled. This postoperative care protocol specifically designed for this chronic experimental model of AMI in rabbits was applied in the same way in the AMI group as in the sham group. It is summarized in Table 1.

#### 2.8.2. Weeks 2, 3, 4, and 5

During these weeks and until the euthanasia of the animals, general physical examination was conducted daily. When necessary, pulmonary and cardiac auscultation was performed. The progression and status of the surgical wound were examined, changing the dressings when required. The digestive functionality was controlled by observing the daily amount of feces, also daily intake was controlled. The body weight of the animals was measured every week (weeks 1–5). Furthermore, in week 5 a transthoracic echocardiographic was performed (Vivid S5, General Electrics Healthcare Madrid, Spain, 10S-RS pediatric probe).

After the 5 weeks of the experimental protocol, 16 rabbits survived (7 in the sham group and 9 in the AMI group). This resulted in a survival rate of 72.7% (77.8% in the sham group and 69.2% in the AMI group). The causes of death of the two animals in the sham group were due to intraoperative and/or postoperative surgical complications, such as respiratory problems.

### 2.9. Arrhythmia Inducibility

**Figure 2:**
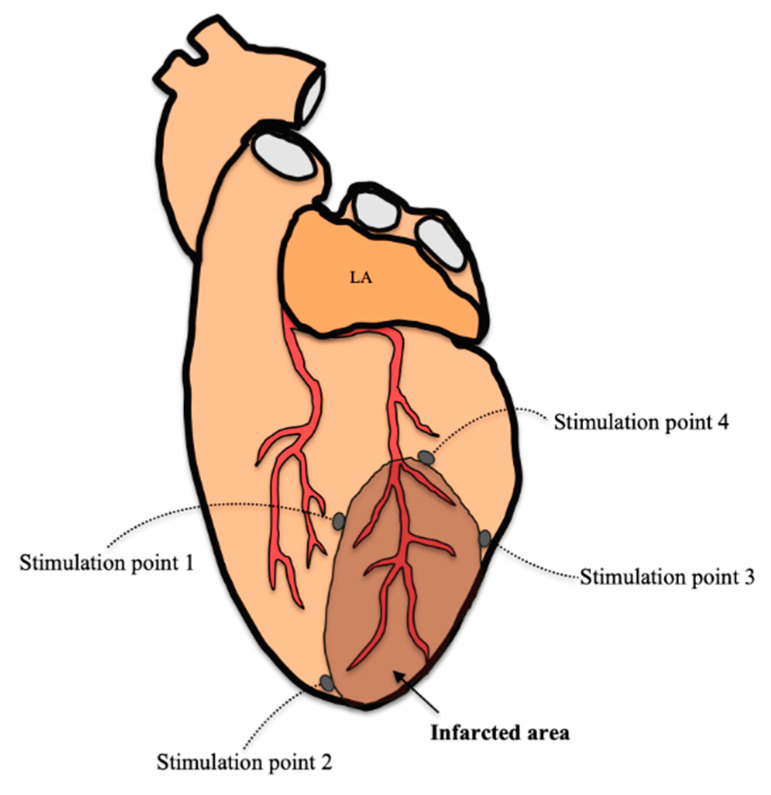
Schematic representation of the distribution of the four ventricular stimulation electrodes (stimulation point) located around the infarcted area. Abbreviations: LA: left atrium.

**Figure 3:**
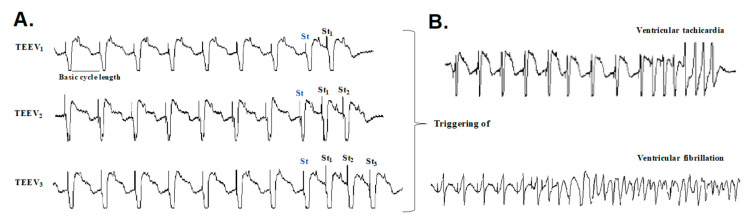
(**A**): An example of the ventricular extrastimulus testing (VEST) applied with up to three extrastimuli (St_1_, St_2_, and St_3_), with St being the last stimulus of the basic series. (**B**): VEST induction of an episode of a self-limiting ventricular tachycardia and a ventricular fibrillation.

After euthanizing the animal intravenously with sodium thiopental, the heart was placed in the Langendorff perfusion system, as previously described [28]. A recording electrode consisting of 256 unipolar stainless-steel electrodes was placed on the epicardium covering the territory irrigated by the occluded coronary artery. The recording and stimulation techniques were similar to those used in other studies [29]. Briefly, the recordings were obtained with a cardiac electrical activity mapping system (MAPTECH, Waalre, Netherlands), and the electrograms were amplified with a gain of 100–300, filtered at a bandwidth of 1–400 Hz, and multiplexed. The sampling frequency was 1 kHz. For ventricular stimulation, four bipolar epicardial electrodes were placed around the infarcted area, as shown in Figure 2. The stimuli were 2-ms rectangular pulses applied at an intensity of twice the diastolic threshold. In both groups (sham and AMI), the inducibility of ventricular arrhythmias was analyzed using ventricular extrastimulus tests (VEST) with two basic stimulation cycles (250 and 150 ms) and up to three extrastimuli for each cycle, an example is seen in Figure 3. The VEST with the 250-ms basic cycle was first applied to each of stimulation point, and the same protocol was repeated for the VEST with the 150-ms basic cycle.

### 2.10. Macroscopic Study

**Figure 4:**
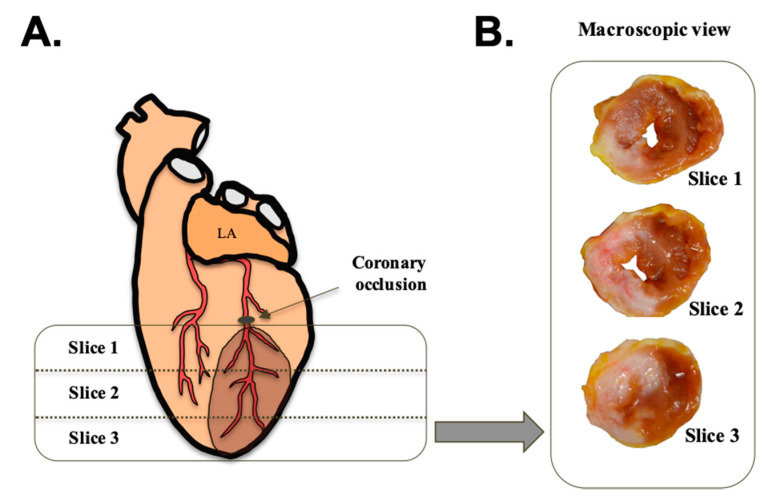
(**A**): A schematic representation of the sections of the heart (slice 1, 2, and 3), located below the coronary ligature. The dark area corresponds to the infarcted area. (**B**): An example of the three sections with thioflavin-S staining without ultraviolet light for macroscopic observation. The necrotic tissue is depicted in each section. Abbreviations: LA: left atrium.

After completing the electrophysiological study, a macroscopic study was performed to quantify the infarct size in relation to the left ventricle and the area at risk. For this purpose, the circumflex coronary artery was transfixed and ligated in the same place that we have reported before (the first third of its epicardial route). Once occluded, the aorta was perfused with thioflavin-S (8 mg/mL). The heart was then sectioned into 3-mm slices using an ad hoc sectioning device for this experimental model. Photographs were taken with room light and ultraviolet light at different wavelengths (254 and 365 nm). Thereafter, the sections were incubated in 2,3,5-triphenyltetrazolium chloride solution (10 g/L) for 20 min in the dark, and different photographs were taken with room light. The images obtained were digitized and manually quantified. For this purpose, the MATLAB 8.4 software package (The MathWorks Inc., Natick, MA, USA) was used. All images were photographed with a ruler positioned next to the myocardial sections as a reference for measurements. Together with the predefined section thickness (3 mm), this allowed calculating the left ventricular myocardial volume. The infarcted tissue was defined as the area of tissue that was not stained with triphenyltetrazolium and expressed as a percentage in relation to the left ventricle. The quantification was performed in three sections (slice 1, 2, and 3), located below the ligature of the coronary occlusion. An example of the location of the sections used is shown in Figure 4.

**Figure 5:**
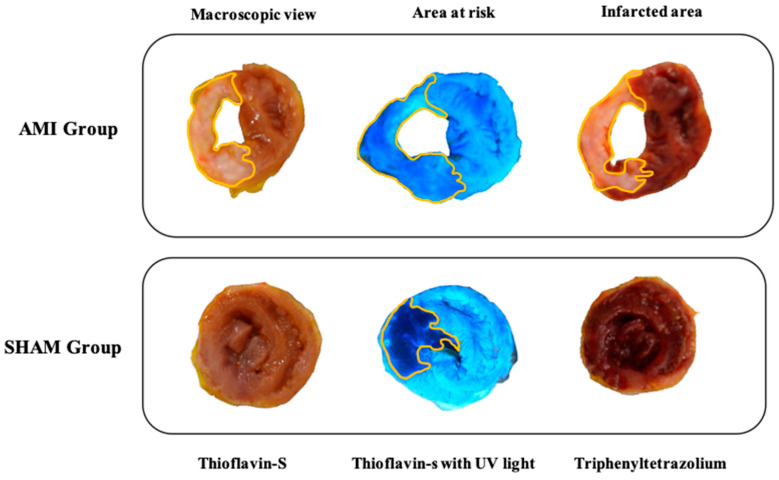
Images from an example of a tissue section for each group (acute myocardial infarction (AMI) and sham) where it is observed the staining of thioflavin-S without ultraviolet (UV) light for macroscopic observation, thioflavin-S with ultraviolet light for determining the area at risk, and triphenyltetrazolium chloride for determining the infarct size.

The area at risk was identified as the area of tissue that did not fluoresce under ultraviolet light. The same experimental protocol was performed in the sham group. Figure 5 shows an example of the different stains used in both groups to determine the infarct size and the area at risk.

### 2.11. Statistics

All values are expressed as mean (standard deviation), unless stated otherwise. The Shapiro-Wilk test was used to assess the normality of data distribution. The association between categorical variables was assessed using the chi-square test. For continuous variables, factorial analysis of variance with one within-subject factor (time: baseline, 5 min, and 60 min after coronary occlusion) and one between-subject factor (group: sham and AMI) was used (IBM S.A. Madrid, Spain, SPSS version 24.0). The results were considered statistically significant when *p* < 0.05.

## 3. Results

### 3.1. Blood Sampling: Biomarkers of Myocardial Necrosis

**Figure 6:**
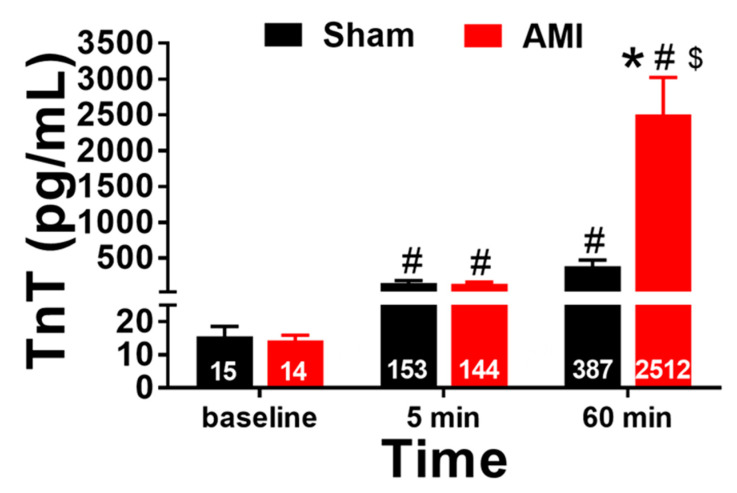
Troponin T (TnT) levels before coronary occlusion (baseline) and after 5 and 60 min of artery reperfusion in the AMI group (n = 9) and at the equivalent times in the sham group (n = 7). * *p* < 0.05 compared with sham, # *p* < 0.05 compared with preocclusion (baseline), and $ *p* < 0.05 compared with 5 min of reperfusion. Error bars = standard error of mean.

Figure 6 shows the TnT levels at each of the studied time points (preocclusion or baseline, 5 min of reperfusion, and 60 min of reperfusion) in the sham and AMI groups. Statistically significant differences were found between the groups (*p* = 0.004, η^2^ = 0.466) and as a function of the measured time (*p* < 0.001, η^2^ = 0.610), as well as in the interaction between both (*p* = 0.002, η^2^ = 0.485). The intergroup comparisons showed that the TnT levels were significantly higher in the AMI group than in the sham group at 60 min of reperfusion (*p* = 0.003, r = 0.692). The intragroup comparisons showed an increase in TnT levels in the AMI group when comparing baseline with 5 min of reperfusion (*p* < 0.001, r = 0.886), baseline with 60 min of reperfusion (*p* < 0.001, r = 0.914), and 5 min with 60 min of reperfusion (*p* < 0.001, r = 0.909). With respect to the sham group, an increase in TnT levels was observed when comparing preocclusion with 5 min of reperfusion (*p* = 0.001, r = 0.899), and between preocclusion and with 60 min of reperfusion (*p* = 0.004, r = 0.878).

**Figure 7:**
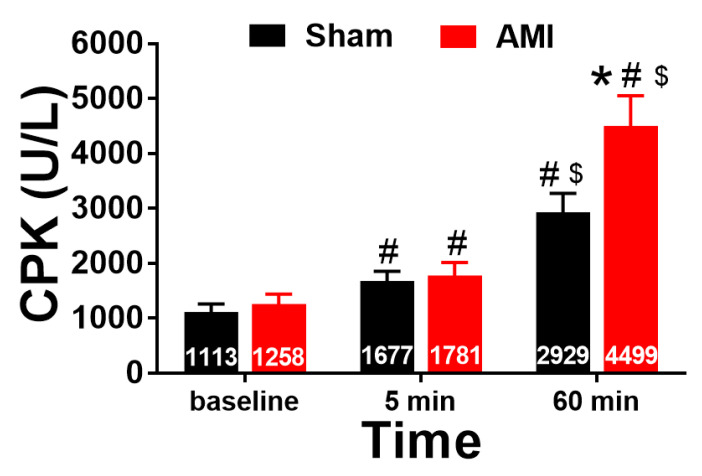
Total creatine phosphokinase (CPK) levels before coronary occlusion (baseline) and after 5 and 60 min of artery reperfusion in the AMI group (n = 9) and at the equivalent times in the sham group (n = 7). * *p* < 0.05 compared with sham, # *p* < 0.05 compared with preocclusion (baseline), and $ *p* < 0.05 compared with 5 min of reperfusion. Error bars = standard error of mean.

With respect to CPK, a major effect of time (Figure 7; *p* = 0.000, η^2^ = 0.759) and the interaction between time and group (*p* = 0.044, η^2^ = 0.236) was found. The analysis of intergroup differences showed that the CPK levels were significantly higher in the AMI group than in the sham group at 60 min of reperfusion (*p* = 0.041, r = 0.515). The intragroup comparisons showed an increase in CPK levels in the AMI group when comparing preocclusion (baseline) with 5 min of reperfusion (*p* = 0.010, r = 0.779), preocclusion with 60 min of reperfusion (*p* < 0.001, r = 0.925), and 5 min with 60 min of reperfusion (*p* < 0.001, r = 0.914). The same trend was observed in the sham group, with an increase in this parameter when comparing preocclusion with 5 min of reperfusion (*p* = 0.015, r = 0.806) and preocclusion with 60 min of reperfusion (*p* = 0.013, r = 0.812).

### 3.2. Echocardiographic Study

**Figure 8:**
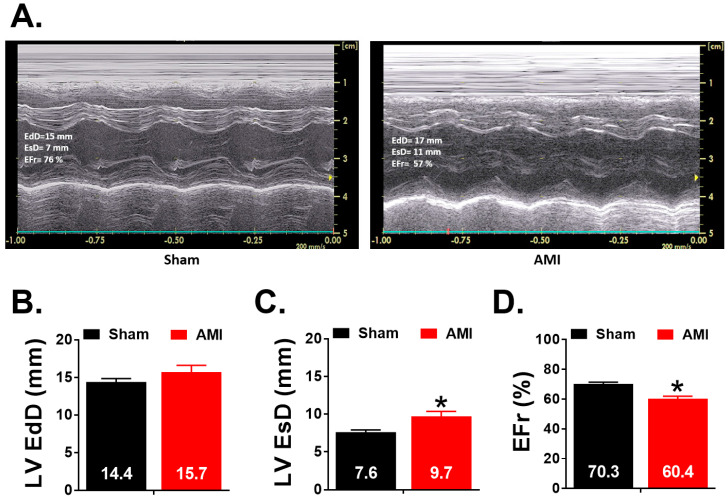
(**A**) M mode recordings of the left ventricle obtained in one experiment of sham group and another of AMI group. (**B**–**D**) Echocardiographic parameters obtained in week 5 in both experimental groups (sham and AMI): left ventricular end diastolic (LV EdD) and systolic (LV EsD) diameters and left ventricle ejection fraction (EFr). * *p* < 0.05 compared with sham group. Error bars = standard error of mean.

Figure 8 shows the echocardiographic parameters related to ventricular size and the ejection fraction obtained in week 5 in both sham and AMI groups. It is also observed two records corresponding to one case from each experimental group. It is observed that the AMI group presents a higher left ventricular end systolic diameter (LV EsD) (*p* = 0.023; r = 0.58) and a lower ejection fraction (EFr) (*p* < 0.001; r = 0.79) than the sham group.

### 3.3. Arrhythmia Inducibility

**Figure 9:**
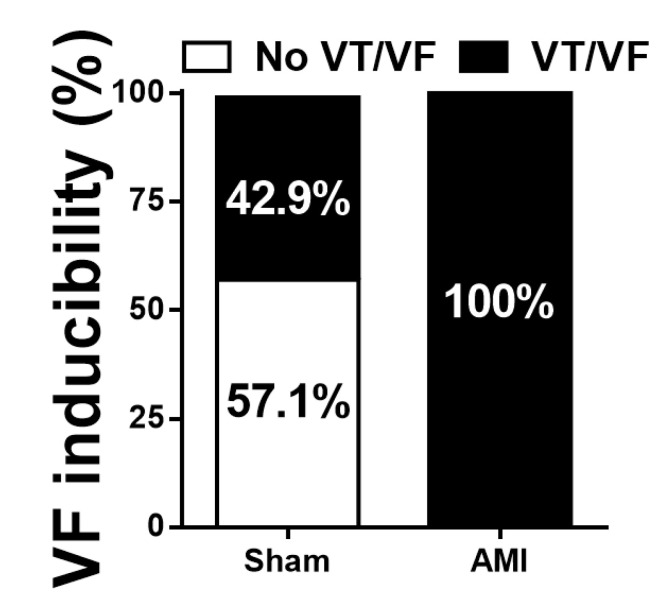
Ventricular tachycardia (VT) and/or ventricular fibrillation (VF) inducibility, and association between groups (sham, acute myocardial infarction) and VT/VF triggering (no VF, VT/VF).

Figure 9 shows the analysis of VT and/or VF inducibility, defined as the ease of triggering the arrhythmia through the application of the VEST, in each group. A statistically significant association was observed between the variables group and ventricular arrhythmia inducibility (*p* = 0.009), with a moderate to high effect size (Phi = 0.655).

### 3.4. Macroscopic Study

**Figure 10:**
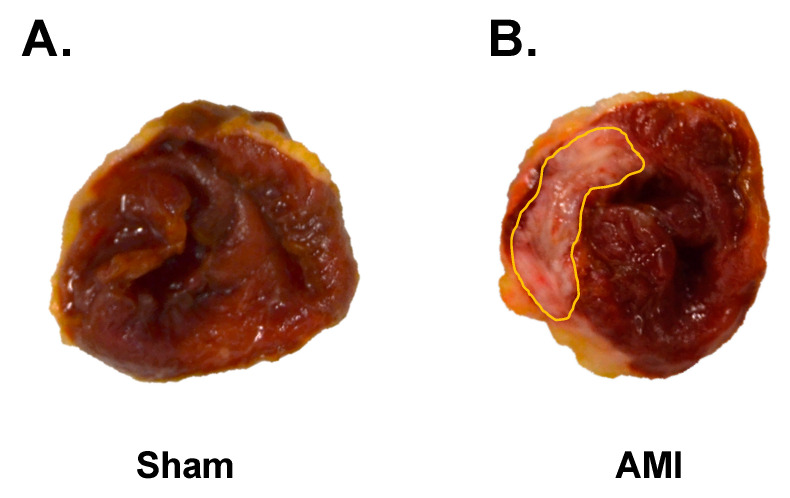
Infarcted area of one experimental case of AMI group (**B**) and an experimental case of the sham group (**A**) where no infarcted area is observed.

The infarct size was quantified using the arithmetic mean of the left ventricle sections, which were located below the coronary ligature. The sections were divided into three slices of equal thickness. Relative to these sections, the infarct size was 39.75 (12.11) %. An example of the infarcted area of an experimental case of the AMI group is shown in Figure 10.

**Figure 11:**
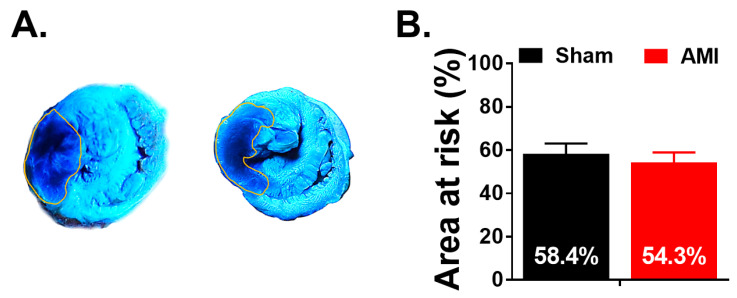
Area at risk. (**A**) shows representative pictures of stained tissue in sham (left) and AMI (**B**) groups. Quantification of the area at risk is depicted in panel B. Sham n = 7, AMI n = 9. Error bars: SEM.

The size of the area at risk of the two groups (AMI and sham) was compared to corroborate the reproducibility of the location of the coronary ligature. The results showed no significant differences between the groups (*p* = 0.557). The percentage of the area at risk was 54.33 (13.99) % and 58.39 (12.54) % in the AMI and sham groups, respectively (Figure 11). An infarct size of 73.16 (21.90) % was obtained in the AMI group when considering the area at risk.

## 4. Discussion

The present study describes the methodological characteristics of the experimental model of chronic (five weeks) myocardial infarction in rabbits. The viability of the model, the care provided to the animals, the characteristics and extent of the lesions, and the inducibility of arrhythmias have been analyzed.

### 4.1. Animal Care

Animal testing is subject to precise regulations and control mechanisms aimed at preventing animal suffering. Compliance with the established requirements starts with the approval of the protocols by the corresponding ethics committees, which includes the analysis of the objectives of the study, and its usefulness to deepen the knowledge on the pathophysiology or the promotion of advances in the diagnosis, treatment, and prevention of diseases, as well as the adequacy of the proposed equipment, techniques, and methods, including anesthesia and/or sedation procedures, surgical procedures, postintervention care, and housing and follow-up conditions [30]. The model used in this study followed the current regulations, and a protocol was developed in which preoperative and postoperative care included pain control, adequate sedation and oxygenation, asepsis, monitoring of vital signs, fluid therapy, and temperature control. The postoperative care considered the prevention of discomfort and pain, control of infections, and correct hydration and nutrition in both the immediate period and the chronic phase.

### 4.2. Viability of the Model

The variables to be considered when selecting the experimental model include its adequacy to achieve the proposed objectives, cost, infrastructure, equipment and personnel needs, ease of handling, housing characteristics, and survival [31]. Several characteristics make rabbits an appropriate model for studying the processes associated with myocardial damage by ischemia/reperfusion [13,32], as follows: their ease of handling; the more reasonable housing conditions and associated costs than when using larger animals; the coronary anatomy and characteristics of the microcirculation, like those of pigs, that are close to those of humans [15,33,34]; and the size of the heart that does not make surgical procedures excessively difficult.

Furthermore, the electrophysiological characteristics of the myocytes of rabbits are closer to those of humans than those of rodents, and arrhythmic complications during the initial follow-up phase are less frequent than in larger animals [35]. The rabbit physiology also has similarities to humans in terms of contractility patterns related to calcium management and myosin heavy chain isoforms [36]. The action potential of rabbit myocardial cells lasts longer than that of rodents, with a spike-and-dome morphology, and repolarization involves K^+^ (I_K+_) ionic currents known as fast (I_Kf_) and slow (I_Ks_) “delayed rectifiers”, in which expression is similar in the ventricular myocardium of rabbits and humans. In the ventricular myocardium of murine models (rat or mouse), the action potential is triangular with a very short duration (around 50 ms) and lacks the repolarization ion currents that are present in the human ventricle, I_Kf_ and I_Ks_ [37,38]. The short duration and triangular shape of mice and rats action potential is mainly determined by the transient outward potassium current (I_to_), in which expression is higher than in rabbits and humans, and by the presence of the ultra-rapidly activating delayed rectifier (IKur) current. With regard to the role of calcium currents, there are also interspecies differences in sarcolemmal and intracellular calcium handling. The shape of the action potential determines the Ca^2+^ influx related to the L-type calcium current (ICaL) time course. On the other hand, the contribution of ICaL and the Na^+^-Ca^2+^ exchanger (NCX) to calcium flux balance and calcium extrusion during cardiac cycles is higher in human and rabbits than in mice and rats, while in the latter the sarcoplasmic reticulum (SR) plays a predominant role and the activity of the SR calcium ATPase (SERCA2), which is involved in the calcium reuptake, is higher [39]. The balance between the activity of SERCA2 and NCX influences the spontaneous Ca^2+^ release and the formation of calcium waves and afterdepolarizations. Susceptibility for the formation de early and delayed afterdepolarizations differs depending on repolarization reserve and on factors, such as sensitivity of the ryanodine receptor, the characteristics of cellular Ca^2+^ fluxes, and the intrinsic heart rate, among other parameters. Differences in calcium handling, action potential restitution and heart size determine a lower susceptibility to electrical alternans phenomena in the experimental models in which mice and rats are used [39]. Moreover, the ventricular mass of the rabbit heart makes it possible to induce ventricular arrhythmias, such as VF [9,40]. However, unlike models using small rodents, the availability of genetically manipulated strains and knockout animals is very limited.

Experimental models of myocardial infarction are subject to complications related to surgical procedures, if applicable, and to other variables, such as the extent of infarction and the appearance of uncontrollable arrhythmias in the acute phase [41]. In the model developed in this study, tracheal intubation was performed without the use of a laryngoscope and the least invasive surgery was performed under total sterile conditions. The monitoring of vital signs throughout the surgery enabled acting promptly when decompensation of any vital sign was observed. A protocol was developed to maintain the animals with an optimal oxygenation level until housing. Once the animals were housed, the provided care ensured survival in an acceptable proportion of the performed experiments.

### 4.3. Induction of Ischemia/Reperfusion

A wide range of experimental techniques are available for modifying or interrupting coronary flow to induce myocardial damage [11,13,14,23,24]. In general, they do not reproduce the mechanisms of occlusion that are present in humans, such as those related to atherosclerosis, development of coronary lesions, and rupture of plaques; however, they can be used to induce acute and chronic occlusions and to study the processes triggered by ischemia and reperfusion, excluding those directly related to vessel obstruction and recanalization. Coronary occlusion can be induced by vessel ligation, embolization, balloon catheter inflation, implantation of stents and intravascular devices, extrinsic compression, implantation of extravascular inflatable rings, and the administration of vasoconstrictor drugs, among other procedures [15,34].

Several authors have noted the similarity between the coronary anatomy of rabbits and that of humans, as the left main artery divides into the left anterior descending artery and the circumflex artery [36]. The anterior descending artery supplies the anterior wall of the heart and most of the ventricular septum, and the circumflex coronary artery initially runs between the left atrium and the left ventricle and supplies the lateral wall of the left ventricle [42]. In the experimental model developed in this study, the circumflex coronary artery was selected because, as other authors have reported, the anterior descending artery is small in rabbits and the apex is supplied by the circumflex artery. Moreover, this artery can be clearly seen on the surface of the anterolateral left ventricle. Occlusion of this branch causes approximately one-third of the ventricle to be ischemic [11].

The duration of the coronary occlusion should be sufficient for irreversible damage and infarction to occur [14]. In the present model, the ligature of the circumflex coronary artery was maintained for 1 h, and irreversible necrosis occurred in the myocardium in which irrigation is subsidiary to this coronary artery, resulting in an area of infarcted tissue. One aspect to consider when studying the processes associated with ischemia together with those associated with reperfusion is how to restore coronary flow after the period of ischemia. The method selected in the present model requires the execution of a median sternotomy but ensures correct visualization of the circumflex artery, adequate selection of the ligature area, and direct observation of the subsidiary area and its function during the ischemic period and immediately after the removal of the obstruction to the flow.

### 4.4. Extent of Myocardial Damage and Characteristics of the Injured Area

The extent and size of the infarcted area is one of the most robust variables for assessing myocardial damage caused by ischemia/reperfusion and the changes that occur after a particular treatment or protective mechanism [11,43]. The procedures used in this study and the complementary staining methods allowed this variable to be directly determined after the experimental protocol has been completed. During the follow-up, monitoring the ventricular function using imaging techniques provides useful information, as does the analysis of markers of myocardial damage in the immediate period after the ischemia/reperfusion procedures. In clinical practice, one of the methods used to establish the diagnosis of myocardial infarction is the detection of biomarkers of myocardial necrosis, such as cardiac TnT and total creatine kinase.

In the developed model, the markers of myocardial damage have only been initially analyzed to detect their existence. The time curve that provides additional information on their scope has not been determined. An increase in TnT levels was observed in the AMI group both after 5 and 60 min of reperfusion. This indicates that, owing to the period of ischemia, cell death mechanisms, such as apoptosis, were initiated in the heart [44], leading to irreversible cardiac tissue damage with loss of sarcolemma cell integrity and release of proteins, including creatine kinase and troponins [45]. The peak troponin value would be more informative than the determinations made in the initial phase of I/R injury. However, the early monitoring of serum cardiac troponin has been useful in proving the existence of myocardial damage. Once the coronary artery ligation is removed, the opening of the artery and the consequent washout that follows reperfusion causes the immediate release of troponin from myocardium into the blood and this rapid release of troponin has been clearly observed in the IM group. In the sham operated group, a significant increase in CPK levels was observed after a time equivalent to 60 min of reperfusion, which was probably related to the surgical procedure, as no significant increase in troponin values was observed after a similar period, and the latter is a selective marker of myocardial damage, whereas CPK is an enzyme that is expressed in multiple tissues, preferably located in the striated musculature [46]. A significant increase in TnT levels was observed in the sham group compared with the baseline values after a time equivalent to 5 min of reperfusion, which may be related to the coronary artery transfixion that was performed in this group to mimic the experimental conditions of the AMI group, although it was performed without proceeding to coronary occlusion in the sham group. Once the suture was placed under the coronary artery the suture was left untied. This procedure can induce mild myocardial damage and a small increase in TnT levels.

The extent of the infarcted area depends on the duration of the ischemia, the area at risk, the existence and magnitude of collateral circulation, and the duration of the reperfusion [11]. The area at risk was defined as the myocardial tissue within the perfusion bed that is distal to the infarction-causing lesion in the related coronary artery. This area at risk included both the ultimately damaged and nonviable cardiac tissue and an area of viable tissue. The existence of a recoverable tissue portion depends, among other factors, on collateral flow and the extent of the damage caused by reperfusion [13]. The characteristics of the coronary circulation and, especially, the existence and extent of collateral circulation determine the characteristics and extent of myocardial damage after the occlusion of one of the coronary arteries [47,48]. Depending on the animal species, important variations exist in collateral circulation, which is very abundant in guinea pigs, moderate in dogs, and very scarce in rabbits and, especially, in pigs [15,33].

Studies in canine models have shown that despite coronary occlusion in the same vessel and in the same anatomical position, infarct size shows great variability, related to differences in the distribution patterns of the coronary arteries and in collateral circulation [49]. It is important to note that in rabbits, as the collateral circulation is scarce and the coronary anatomy is rather constant, there is less variability in infarct size between different experimental cases and the results are more reproducible with respect to its extent.

In the model used in this study, both the scarce collateral circulation and the controlled location of the ligature and occlusion time have allowed uniform infarcted area values to be obtained, which have also corresponded to a high proportion of the area at risk determined by thioflavin-S perfusion. In each section of the preparation, vital staining with 2,3,5-triphenyltetrazolium chloride has enabled the identification of viable tissue, to differentiate it from necrotic tissue and to quantify the proportion of the latter in relation to the area of the section and to the area at risk identified by thioflavin-S perfusion. In the sham operated group, in which the suture positioned under the circumflex artery during the surgical procedure was untied, no infarcted area was observed in the area irrigated by the circumflex artery. The determination of the area at risk after conducting the electrophysiological study has allowed us to analyze its extension, regardless of whether the artery had been temporarily occluded (AMI group) or not (sham group), and the reproducibility of the procedure.

### 4.5. Arrhythmia Inducibility

Arrhythmias related to ischemic heart disease respond to various mechanisms. The acute phase of myocardial infarction is characterized by great instability that results in episodes of VF and sudden death in patients. Factors, such as the extent of the ischemic area, the characteristics of collateral flow, the speed of the establishment of the coronary irrigation deficit, the activity of the autonomic nervous system, and the presence of the reperfusion phenomenon, play a role [8,18]. In chronic models of myocardial damage due to ischemia/reperfusion, there are other factors, including the structural alterations inherent to the healing process, that determine the characteristics of the substrate that facilitates the appearance of certain arrhythmias, such as VT and/or VF. The electrophysiological heterogeneity and the existence of obstacles and unidirectional blockade areas favor reentry mechanisms. The model used in this study allows the use of high-resolution mapping techniques, as well as the analysis of responses to programmed stimulation, including the inducibility of arrhythmias and the determination of basic parameters, such as refractory periods, conduction velocity, and wavelength of the activation process, which can be correlated with arrhythmia inducibility [9,40]. Compared with murine models, those based on the use of rabbits show more similarities with the human heart. This is because the repolarization process and the ionic currents involved are different in murine models, as the late rectifying potassium current is not relevant, which are characteristics that contribute to the limitations inherent to the small size of the hearts in murine models that make it difficult to trigger arrhythmias.

The present study showed a significant association between the experimental group and the triggering of VT and/or VF. In the AMI group, VT and/or VF were triggered by programmed stimulation in all cases. The inducibility of ventricular arrhythmias has been studied in the chronic phase of infarction [50,51,52], a phase in which dead cardiomyocytes are replaced by collagen, leading to scar formation. This scar acts as an inexcitable anatomic obstacle that can impair the normal progression of the excitation process and increase electrophysiological heterogeneity, thus favoring the appearance of arrhythmias [8,53]. The increase in electrophysiological heterogeneity with the appearance of conduction blocks and the spatial dispersion of repolarization promotes the appearance of reentry processes and arrhythmias caused by this mechanism [54]. In the sham operated group, the triggering of arrhythmias was observed in some cases. This may be due to the intensity of the ventricular stimulation protocol used in this study, as up to three extrastimuli were applied in the VEST, with two different basic cycles and in four different stimulation areas.

## 5. Conclusions

This study presents a reproducible chronic experimental model of myocardial infarction in which the extent and characteristics of the lesions enable the study of the vulnerability to develop ventricular arrhythmias as a consequence of the remodeling process that occurs during cardiac tissue repair after the acute episode.

## Figures and Tables

**Table 1:** Summary of the postoperative care protocol designed and applied during the first week after surgery. Abbreviations: IV, intravenous; SC, subcutaneous.

Week 1				
**Postoperative** **care**	**Day 0**	**Day 1**	**Day 2**	**Day 3**
	**Surgery**	Heat with infrared light	Heat with infrared light	Heat with infrared light
		Fluid Therapy (IV)	Fluid Therapy (IV)	Fluid Therapy (IV)
		Analgesic treatment (SC) 0.03 mg/Kg/6 h	Analgesic treatment (SC) 0.03 mg/Kg/6 h	Analgesic treatment (SC) 0.03 mg/Kg/6 h
		Anti-inflammatory treatment (SC) 0.2 mg/Kg/24 h	Anti-inflammatory treatment (SC) 0.2 mg/Kg/24 h	Anti-inflammatory treatment (SC) 0.2 mg/Kg/24 h
		Antibiotic treatment (SC) 10 mg/Kg/24 h	Antibiotic treatment (SC) 10 mg/Kg/24 h	Antibiotic treatment (SC) 10 mg/Kg/24 h
		Control of digestive functionality	Control of digestive functionality	Control of digestive functionality
		General physical examination	General physical examination	General physical examination
	**Day 4**	**Day 5**	**Day 6**	**Day 7**
	Heat with infrared light	-	-	-
	-	-	-	-
	Analgesic treatment: osmotic pump: 0.03 mg/Kg rate of 10 µL/h	Analgesic treatment: osmotic pump: 0.03 mg/Kg rate of 10 µL/h	Analgesic treatment: osmotic pump: 0.03 mg/Kg rate of 10 µL/h	Analgesic treatment: osmotic pump: 0.03 mg/Kg rate of 10 µL/h
	Anti-inflammatory treatment (SC) 0.2 mg/Kg/24 h	Anti-inflammatory treatment (SC) 0.2 mg/Kg/24 h	Anti-inflammatory treatment (SC) 0.2 mg/Kg/24 h	Anti-inflammatory treatment (SC) 0.2 mg/Kg/24 h
	Antibiotic treatment (SC) 10 mg/Kg/24 h	Antibiotic treatment (SC) 10 mg/Kg/24 h	Antibiotic treatment (SC) 10 mg/Kg/24 h	Antibiotic treatment (SC) 10 mg/Kg/24 h
	Control of digestive functionality	Control of digestive functionality	Control of digestive functionality	Control of digestive functionality
	General physical examination	General physical examination	General physical examination	General physical examination
